# Inhibition of SRC-3 as a potential therapeutic strategy for aggressive mantle cell lymphoma

**DOI:** 10.21203/rs.3.rs-2653461/v1

**Published:** 2023-03-10

**Authors:** Imani Bijou, Yang Liu, Dong Lu, Jianwei Chen, Shelby Sloan, Lapo Alinari, David M. Lonard, Bert W. O’Malley, Michael Wang, Jin Wang

**Affiliations:** Baylor College of Medicine; The University of Texas MD Anderson Cancer Center; Baylor College of Medicine; Baylor College of Medicine; The James Cancer Hospital and Solove Research Institute, The Ohio State University; The James Cancer Hospital and Solove Research Institute, The Ohio State University; Baylor College of Medicine; Baylor College of Medicine; The University of Texas MD Anderson Cancer Center; Baylor College of Medicine

**Keywords:** Mantle cell lymphoma, SRC-3, drug resistance, antitumor activity

## Abstract

Mantle cell lymphoma (MCL) is a heterogeneous disease with a poor prognosis. Despite years of research in MCL, relapse occurs in patients with current therapeutic options necessitating the development of novel therapeutic agents. Previous attempts to pharmacologically inhibit SRC-3 show effectiveness *in vivo* and *in vitro* in other B cell lymphomas, and previous studies have shown that SRC-3 is highly expressed in the lymph nodes of B cell non-Hodgkin’s lymphoma patients. This suggests that SRC-3 may play a role in the progression of B cell lymphoma and that the development of selective SRC inhibitors should be investigated. This study aimed to investigate novel SRC-3 inhibitors, SI-10 and SI-12, in mantle cell lymphoma. The cytotoxic effects of SI-10 and SI-12 were evaluated in a panel of MCL cell lines *in vitro* by resazurin assay. The *in vivo* efficacy of SI-10 was confirmed in two ibrutinib-resistant models: an immunocompetent disseminated A20 mouse model of B-cell lymphoma and a human PDX model of MCL. SI-10 treatment resulted in dose-dependent cytotoxicity in a panel of MCL cell lines *in vitro*. Notably, SI-10 treatment also resulted in a significant extension of survival *in vivo* with low toxicity in both ibrutinib-resistant murine models. We have investigated SI-10 as a novel anti-lymphoma compound via the inhibition of SRC-3 activity. These findings indicate that targeting SRC-3 should be investigated in combination with current clinical therapeutics as a novel strategy to expand the therapeutic index and to improve lymphoma outcomes.

## Introduction

Non-Hodgkin lymphoma (NHL) is the most commonly occurring hematological malignancy containing a variety of subtypes. Mantle cell lymphoma (MCL) is an aggressive and incurable subtype of B-cell-NHL accounting for 4% of all lymphomas and resulting in a median survival of 8–12 years [1, 2]. Most patients present with advanced-stage disease, and current therapies, which include anti-CD20 monoclonal antibodies, autologous stem-cell transplantation, immunochemotherapy, and targeted therapy, have been unable to eradicate MCL resulting in almost universal relapse [3]. Given the current efficacy of standard lymphoma treatment regimens, therapies have been investigated targeting B-cell receptor pathway signaling or apoptotic pathway signaling to treat relapsed/refractory disease [1]. The BTK inhibitor ibrutinib and some of its analogs have resulted in high overall response rates and FDA approval. However, resistance and relapse still occur resulting in treatment regimens combining other targeted therapies and necessitating approaches with differing mechanisms of action to those clinically approved [4–6].

The steroid receptor coactivator (SRC) family contains three members, SRC-1, SRC-2, and SRC-3 [7]. These transcriptional coactivators function through interactions with nuclear receptors which then recruit additional proteins to form multi-subunit transcriptional complexes that promote transcriptional activity. Additionally, SRCs have been shown to coactivate various non-nuclear receptor transcriptional factors such as NF-kB, AP-1, and E2F1, and interact with the CBP/p300 coactivators [4]. The dysregulation of epigenetic modulators is an initiator of carcinogenesis, and SRC-3 overexpression has been associated with malignancy in breast, lung, and prostate cancer [8–10]. SRC-3 has also been shown to promote tumor growth through involvement in pathways regulating cell cycle, apoptosis, drug resistance, migration, and invasion [11].

The role of SRC-3 in MCL yet to be well defined, but previous studies show that SRC-3 is highly expressed in B-cell NHL models. Pharmacological inhibition of SRC-3 with gambogic acid reduced tumor growth of diffuse large cell b cell lymphoma (DLBCL) models *in vitro* and *in vivo* through histone deacetylation and downregulation of multiple oncoproteins such as Bcl-2, cyclin D3, Bcl-6, and c-Myc [12]. Additionally, single-cell transcriptomic data suggest that SRC-3 is most highly expressed in B cells and plasma cells, suggesting a potential undiscovered role of SRC-3 in B cell receptor signaling [13].

In collaboration with Drs. O’Malley and Lonard’s groups, we identified SI-2 as a potent SRC-3 inhibitor capable of decreasing SRC-3 protein levels and inhibiting breast cancer cell proliferation *in vitro* and tumor growth *in vivo* [14]. More recently, SI-2 was optimized for more favorable pharmacokinetic properties by introducing of up to three fluorine atoms generating the analogs SI-10 and SI-12. These analogs have been shown to exert potent anti-cancer activity with minimal cardiotoxicity in breast cancer models[15]. In this study, we aim to investigate the anti-cancer effect of new SRC-3 inhibitors *in vitro* and *in vivo* for the treatment of MCL.

## Materials And Methods

### CELL CULTURE

Cells were grown in RPMI-1640 supplemented with 10% fetal bovine serum (FBS) and 1% penicillin-streptomycin (10000 U/mL) and cultured at 37 °C in a humidified incubator at 5% CO_2_ for all experiments. Human MCL cell lines (JeKo-1, Mino, Maver-1, Z-138) were gifted from Dr. Michael Wang’s lab (MD Anderson). The murine-derived A20 cells are from ATCC and were maintained in RPMI-1640 supplemented with 0.05 mM 2-mercaptoethanol, 10% FBS, and 1% penicillin-streptomycin.

### CELL VIABILITY ASSAY

Cells were seeded in 96 well plates at a density of 5000–8000 cells per well. The next day cells were treated with serially diluted SRC-3 inhibitors for 48 h along with DMSO control. Cell viability was measured using the Alamar Blue assay. Resazurin was added at 10% of well volume and incubated with cells for 4 hours. Fluorescence was measured at excitation/emission 544/590 nm. Viability was calculated by plotting viability relative to control. The IC_50_ values for compounds were calculated based on the Hill-Slope equation and analyzed in GraphPad Prism 9 (GraphPad Software, Inc., San Diego, CA, USA).

#### *IN VIVO* EXPERIMENTS

##### Therapeutic efficacy of SI-10 in A20 lymphoma mice

To establish an immunocompetent lymphoma model, 1 × 10^6^ A20 cells were injected via the tail vein into female BALB/c mice. A week later, mice were treated with 1 mg/kg/day SI-10 or vehicle control via intraperitoneal injection for 5 weeks. Animals were monitored daily and sacrificed if they displayed hind limb paralysis or a 20% reduction in body mass.

##### Therapeutic efficacy of SI-10 in ibrutinib-resistant MCL PDX mice

To establish a xenograft model, 5 million cells were injected subcutaneously into NSG mice (6-week-old). After the formation of palpable tumors, mice were treated orally 5 days per week with SI-10 50 mg/kg, ibrutinib 50 mg/kg, or vehicle. Tumor size and body weight were measured 3 times a week.

All animal protocols were approved by the IACUC at Baylor College of Medicine and MD Anderson Cancer Center.

### STATISTICAL ANALYSIS

All statistical graphs are constructed using Prism 9 (GraphPad Software, Inc.). P < 0.05 was considered statistically significant.

## Results

### SRC-3 INHIBITORS REDUCE THE PROLIFERATION OF VARIOUS MCL CELL LINES

**Figure 1:**
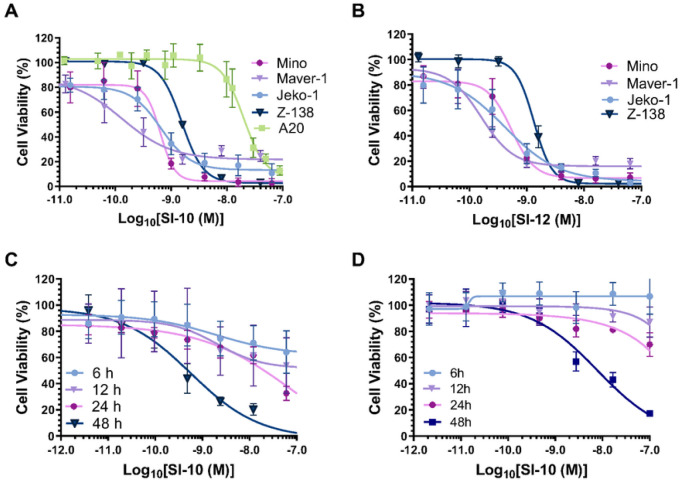
MCL cell lines were treated with SRC-3 inhibitors A) SI-10 or B) SI-12 at various concentrations for 48 h and cell viability was determined using the resazurin assay C) Mino D) Jeko-1 growth inhibition with SI-10 (0–1 mM) for 6,12,24, or 48h

Previous studies have shown SRC-3 overexpression to be pivotal in many solid tumors, but the significance of SRC-3 in blood cancer tumors was undetermined [8–10]. Recently, a study showed clinical SRC-3 overexpression in the lymph nodes of B-cell NHL patients [12]. To evaluate the *in vitro* anti-lymphoma activity of SI-10 and SI-12, four MCL lines and one murine lymphoma line were treated with either SRC-3 inhibitor SI-10 or SI-12 at concentrations ranging from 0–2 μM. Cell viability was assessed using the Alamar Blue assay after 48 hours. After treatment, both SI-10 and SI-12 significantly inhibited the growth of lymphoma in a dose-dependent manner. IC_50_ values were calculated at the low nanomolar level for both SI-10 and SI-12 (Fig. 1A and B). Comparing the IC_50_ values of the two drugs, they both demonstrated similar efficacy in the human MCL cell lines. The murine A20 B cell lymphoma line showed the most resistance to SI-10 with an IC_50_ of 20 nM. Cell viability was also measured at different treatment times 12, 24, and 48 h at concentrations up to 2 μM. Viability also decreased in a time-dependent manner (Fig. 1C and D).

#### SRC-3 INHIBITORS OVERCOME DRUG RESISTANCE IN VITRO

The first-line treatment of MCL includes chemotherapy and the anti-CD20 monoclonal antibody rituximab, but given that so many patients relapse, targeted therapies are frequently offered as a second-line option. Current targeted therapy for MCL includes the FDA-approved BTK inhibitor ibrutinib and the Bcl-2 inhibitor venetoclax currently in clinical trials. B–cell receptor (BCR) signaling is highly upregulated in B-cell malignancies, and inhibition of BTK leads to durable clinical responses in MCL [16]. Additionally, Bcl-2 regulates the intrinsic mitochondrial apoptotic pathway where its overexpression results in mitochondrial outer membrane permeabilization through the interplay of pro- and anti-apoptotic Bcl-2 family proteins [17]. Despite the clinical efficacy of both drugs, resistance to venetoclax and ibrutinib is still of concern as most patients progress on both drugs [18, 19]

**Figure 2:**
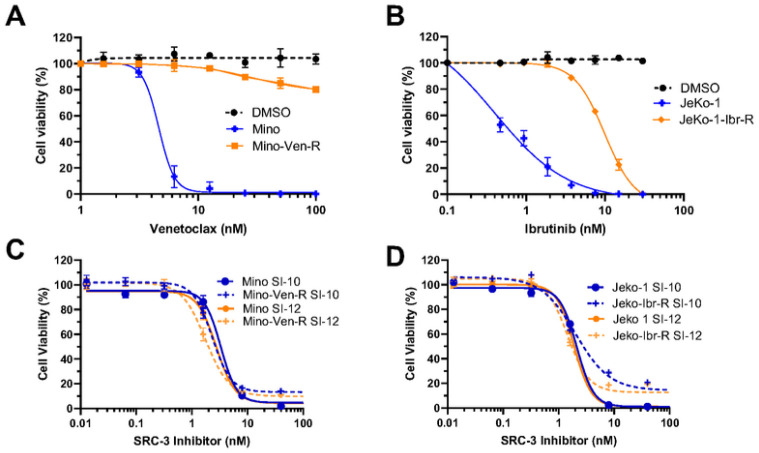
Mino parental and Mino venetoclax resistant cells were treated with various concentrations of venetoclax. B) Jeko-1 parental and Jeko-1 ibrutinib resistant cells were treated with ibrutinib at various concentrations. C) Mino parental and resistant cells were treated with SI-10 and SI-12. D) Jeko-1 parental and resistant cells were treated with SI-10 and SI-12

High levels of SRC-3 have been associated with resistance to chemotherapy and targeted therapies in cancer. SRC-3 has been associated with resistance to chemotherapy and targeted therapies in cancer models. Targeting SRC-3 with bufalin resulted in reduced polarization of pro-tumorigenic M2 macrophages by decreasing MIF expression in chemo-resistant colon cancer models [20]. Additionally, siRNA downregulation of SRC-3 reverses tamoxifen resistance in endocrine-resistant, HER2-positive breast cancer cells [21]. We next investigated if SRC-3 inhibitors were effective in drug-resistant Jeko-1 and Mino cells given their efficacy in the parental cell lines. Venetoclax and ibrutinib-resistant mantle cell lymphoma lines were established in Dr. Michael Wang’s lab by exposing the cells to stepwise dose increases of the drug [22, 23]. Compared to the parental cells, both venetoclax and ibrutinib-resistant cells showed reduced sensitivity when treated with their respective inhibitors (Fig. 2A and B). The efficacy of both SRC-3 inhibitors, SI-10 and SI-12, was then evaluated in the resistant lines. The SRC-3 inhibitors maintained nanomolar IC_50_ in venetoclax-resistant Mino cells and ibrutinib-resistant Jeko-1 cells (Fig. 2. C and D). This suggests that SRC-3 inhibitors may be effective in drug-resistant models of MCL.

### Si-10 Overcomes Ibrutinib Resistance In Pdx Mouse Models

**Figure 3:**
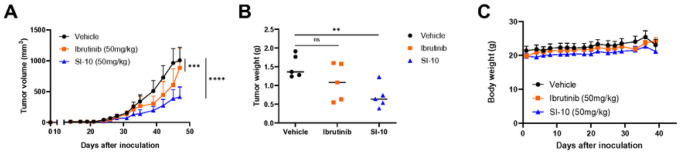
SRC-3 treatment decreased tumor growth in a Jeko-1 ibrutinib resistant mouse model. A) Volumes of tumors treated with SI-10 (50 mg/kg), ibrutinib (100mg/kg) or vehicle B) Weight of tumors treated with SI-10 (50 mg/kg), ibrutinib (100mg/kg) or vehicle before sacrificing C) The weight of each mouse was measured 3 times a week

Most MCL patients eventually relapse; therefore it is critical to find drugs that are effective even after primary treatment. The MCL lines screened had varying sensitivities to ibrutinib, but all had low nanomolar IC_50_ for our SRC-3 inhibitors. To evaluate the efficacy of SI-10 against ibrutinib resistance, we established a PDX mouse model with ibrutinib resistance in 6-week-old NSG mice by subcutaneous injection of ibrutinib-resistant Jeko cells. Mice were treated for 7 weeks five times a week with either vehicle, ibrutinib (50 mg/kg), or SI-10 (50 mg/kg) via oral gavage. Here we see reduced tumor volume and tumor weight after treatment between SI-10 and vehicle, and we also see an improvement compared to ibrutinib treatment (Fig. 3A and B). Additionally, no significant body weight changes were observed throughout the treatment course (Fig. 3C). This suggests that SI-10 could be beneficial against ibrutinib-resistant MCL.

### Si-10 Inhibition Extends Survival In A Syngeneic Tumor Model

**Figure 4:**
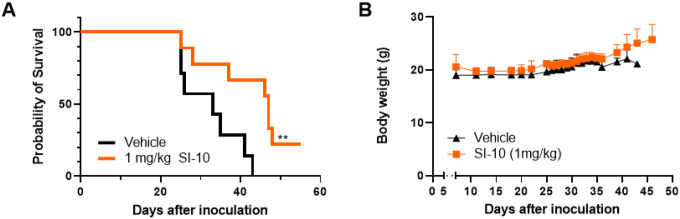
SRC-3 treatment prolonged survival of A20-Balb/c mice. Balb/c mice were injected with 1 × 10^6^ A20 cells intravenously. Treatment started on day 7. A) Kaplan-Meier survival plot reflecting time to lethal tumor burden. Based on the log-rank test, there are significant differences between the treated group and the control (P < 0.05). B) Body weight of all groups were measured 5 days a week

We next investigated SI-10 in an A20 immunocompetent syngeneic mouse model of B cell lymphoma. With increasing knowledge of the tumor microenvironment, preclinical studies show that the TME plays a significant role in drug resistance to current therapies [24]. The A20 model exhibits clinical characteristics of MCL in vivo, including infiltration of the bone marrow and liver, and enlargement of the spleen [25]. This model has also been shown to exploit evasive immune mechanisms across tumor progression. Specifically, injection of A20 cells has been shown to induce an expansion of regulatory T cells [26]. Tumors generate an immunosuppressive environment to maintain optimal growth, and recent studies show SRC-3 may contribute to this suppressive environment. Inhibition of SRC-3 may act dually, reducing cancer cell growth and blocking the activity of suppressive immune cells like T regulatory cells (Tregs); this has been shown *in vivo* in breast cancer models [27, 28]. We evaluated if SRC-3 inhibition would still be effective in an immunosuppressive environment. A20 cells were injected via the tail vein of Balb/c mice. A week later, mice were randomized into two groups (n = 7) to receive SI-10 at 1 mg/kg vehicle. Mice received daily intraperitoneal injections over 7 weeks with SI-10 or vehicle. During the treatment period, mice were evaluated for gross abnormalities associated with the model and sacrificed according to protocol. As shown, mice treated with 1 mg/kg of SI-10 have a median survival of 47 days, significantly extending survival compared to the vehicle-treated mice with a median survival of 33 days (Fig. 4A). No noticeable side effects were observed during treatment and body weight was not significantly affected (Fig. 4B).

Figure 4 SRC-3 treatment prolonged survival of A20-Balb/c mice. Balb/c mice were injected with 1 × 10^6^ A20 cells intravenously. Treatment started on day 7. A) Kaplan-Meier survival plot reflecting time to lethal tumor burden. Based on the log-rank test, there are significant differences between the treated group and the control (P < 0.05). B) Body weight of all groups were measured 5 days a week

### CHRONIC TREATMENT with SI-10 IS WELL TOLERATED

**Figure 5:**
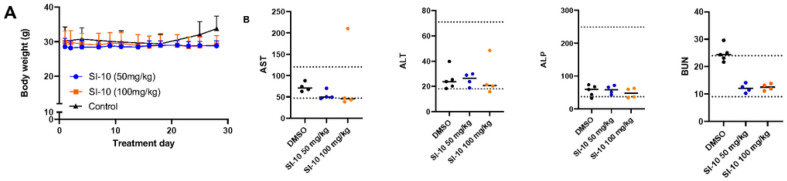
SRC-3 inhibitor treatment in normal mice show little to no toxicity after treatment for 28 days A) Body weight of all groups were measured 5 days a week. B) Blood serum levels of clinical markers related to kidney and liver failure. Dotted lines represent upper and lower limits based on reference values

To evaluate chronic toxicity, ICR mice were treated daily with 50 mg/kg or 100 mg/kg SI-10 for 4 weeks and serum chemistry analysis was performed. The highest dose is 2 times that used in the mouse xenograft model above. Throughout the treatment course, we see no significant body weight fluctuations (Fig. 5A). Compared to literature reference values and the control, no obvious difference was observed in the levels of liver enzymes AST and ALT (Fig. 5B). Only one mouse in the 100 mg/kg group had high AST levels. Regarding kidney function, BUN levels were observed to be comparable to that of the reference range, although a bit lower than the control.

## Discussion

Much research has been done exploring the role of SRC-3 in carcinogenesis through the regulation of oncoproteins and transcription factors in solid tumors, but the role of SRC-3 in blood cancers is less clear. SRC-3 is overexpressed in the lymph nodes of other B cell malignancies, but its role in MCL is not well characterized. MCL patients frequently undergo relapse to current therapies, necessitating novel treatment modalities to improve outcomes. This study demonstrates the therapeutic potential of SRC-3 inhibitor SI-10 as a treatment for *in vitro* and *in vivo* models of mantle cell lymphoma. SI-10 also exhibited inhibitory effects in drug-resistant models of MCL.

Previous data surrounding SRC-3 and B-cell malignancies is a bit conflicting regarding the possibility of SRC-3 as a tumor suppressor or oncogene. SRC-3 overexpression has been shown to contribute to other aggressive non-Hodgkin B cell lymphomas. Inhibition of SRC-3 in DLBCL with gambogic acid resulted in cell cycle arrest and apoptosis in B cell NHL lines. Treatment was also associated with the downregulation of DLBCL oncoproteins, including Bcl-2, Bcl-6, c-Myc, and NF-kB [12]. Unlike gambogic acid, SRC-3 has been confirmed to be a target of SI-10 [15]. Gambogic acid has been shown to target multiple cancer-related proteins in addition to SRC-3, including Bcl-2 family proteins, the proteasome, and topoisomerase IIa exhibiting polypharmacology [29–31]. Despite efficacy with pharmacological inhibition of SRC-3, complete amelioration of SRC-3 via knockout *in vivo* shows the specific induction of extreme lymphoproliferation of both T and B cells, eventually progressing into B-cell lymphoma with age. Interestingly, the *in vivo* studies showed no effect on cell proliferation and apoptosis in other tissue types in the SRC-3 knockout mice [32].

We demonstrate here that SI-10 is a potent anti-tumor small molecule for the treatment of MCL *in vitro* and *in vivo*. SI-10 exhibits low nanomolar efficacy in a panel of MCL cell lines. SRCs have been shown to regulate a multitude of pathways involved in cancer progression and metastasis, including known targets of MCL. Several signaling molecules have been implicated in MCL pathogenesis, including PI3K/AKT, NF-kB, and Bcl-2 [33]. SRC-3 has previously been shown to coactivate many of the targets important in MCL pathogenesis in other cancer types [10, 34, 35]. Many of these same pathways are also implicated in ibrutinib resistance. Ibrutinib-resistant cells activate BCR signaling through the PI3K/AKT pathway and NF-kB signaling, and maintain cell cycle progression through cyclin D1 to undergo primary resistance [19]. These findings suggest that SRC-3 signaling may be important for MCL cell survival and drug resistance through the possible coactivation of these targets.

In addition to its efficacy *in vitro*, SI-10 has demonstrated survival extension in an immunocompetent lymphoma model and a human PDX model both resistant to ibrutinib. The role of SRC-3 in cancer drug resistance is context-dependent, but previously SRC-3 overexpression has been shown to contribute to Herceptin resistance in ERBB2 overexpressing breast cancer cells [36]. Given the high rate of relapse against current MCL treatments, overcoming drug resistance is especially exciting and warrants further study. SI-10 also has a good safety pro le. In the studies performed here, mice in both treated and control groups exhibited normal behavior and minimal body weight loss. SI-10 treatment was well tolerated even at 50 times the dose tested for survival extension.

In conclusion, SI-10 is a small molecule SRC-3 inhibitor that can inhibit MCL *in vitro* and *in vivo* and overcome ibrutinib resistance. With further studies, SI-10 may be a promising therapeutic candidate for ibrutinib-resistant MCL.
